# No Light, No Germination: Excitation of the Rhodospirillum centenum Photosynthetic Apparatus Is Necessary and Sufficient for Cyst Germination

**DOI:** 10.1128/mBio.03619-20

**Published:** 2021-03-16

**Authors:** Nandhini Ashok, Kuang He, Carl E. Bauer

**Affiliations:** a Department of Biology, Indiana University, Bloomington, Indiana, USA; b Department of Molecular and Cellular Biochemistry, Indiana University, Bloomington, Indiana, USA; University of Washington

**Keywords:** Gram-negative cyst cell germination, *Azospirillum* clade, photosynthesis, light germinate

## Abstract

Rhodospirillum centenum is a Gram-negative alphaproteobacterium that is capable of differentiating into dormant cysts that are metabolically inactive and desiccation resistant. Like spores synthesized by many Gram-positive species, dormant R. centenum cysts germinate in response to an environmental signal, indicating that conditions favor survival and proliferation. Factors that induce germination are called germinants and are often both niche and species specific. In this study, we have identified photosynthesis as a niche-specific germinant for *R. centenum* cyst germination. Specifically, excitation of wild-type cysts suspended in a nutrient-free buffer with far-red light at >750 nm results in rapid germination. This is in stark contrast to mutant strains deficient in photosynthesis that fail to germinate upon exposure to far-red light under all assayed conditions. We also show that photosynthesis-induced germination occurs in a carbon- and nitrogen-free buffer even in strains that are deficient in carbon or nitrogen fixation. These results demonstrate that photosynthesis not only is necessary for germination but is itself sufficient for the germination of *R. centenum* cysts.

## Abstract (precis)

Environmental cues that signal Gram-positive spores to germinate (termed germinants) have been identified for several *Bacillus* and *Clostridium* species. These studies showed that germinants are niche and species specific.

## INTRODUCTION

**FIG 1:**
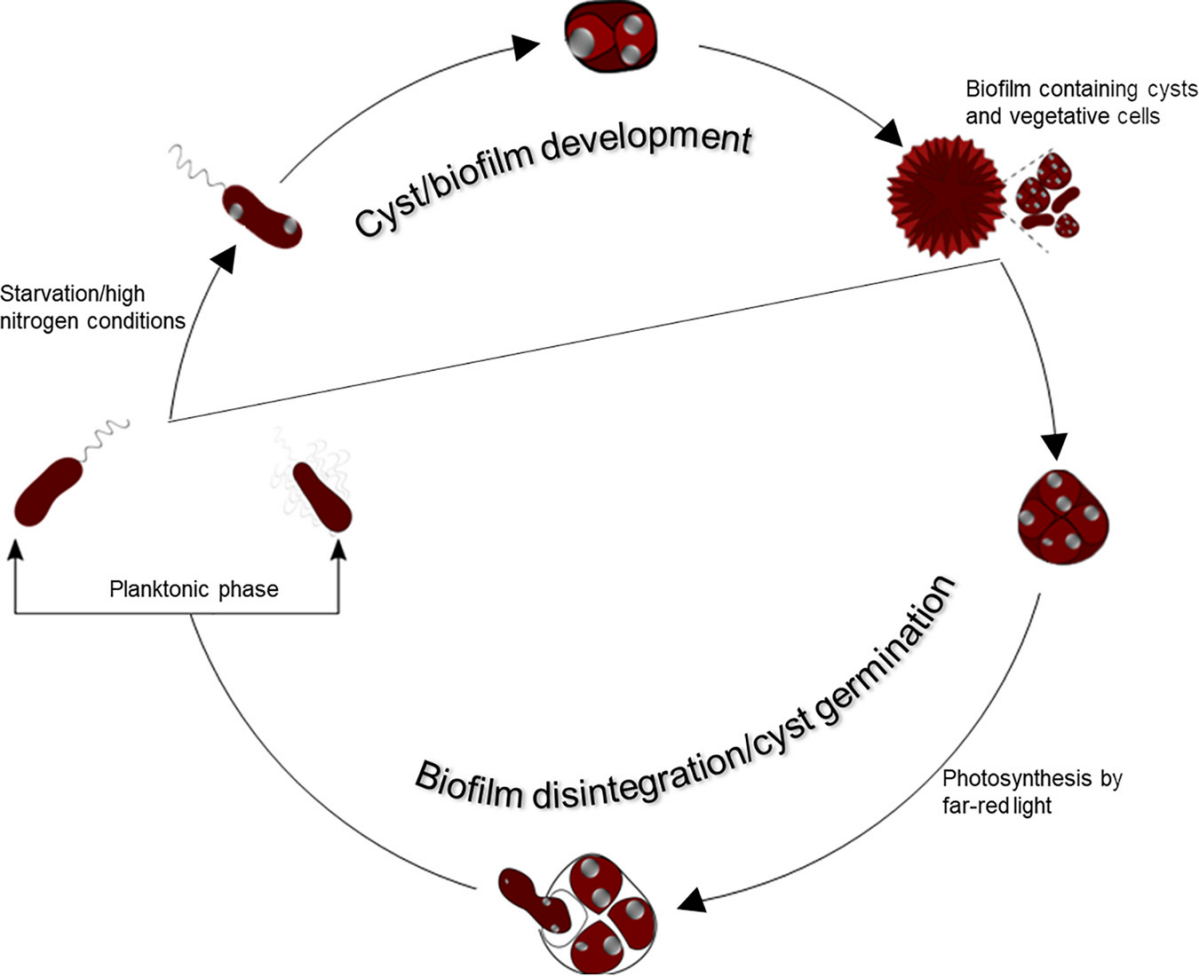
The *R. centenum* life cycle. The upper clockwise portion of the cycle shows the developmental pathway where vegetative cells differentiate into cysts. This process involves the production of large quantities of polyhydroxybutyrate (PHB) storage granules, depicted as internal gray spots. Clusters of dormant cells are also covered with a thick polysaccharide layer called the exine coat. The lower portion of the developmental cycle shows cysts that are undergoing the process of germination.

Most purple photosynthetic bacteria exhibit a wide range of growth capabilities, such as growth under dark heterotrophic conditions as well as growth under photosynthetic conditions with or without the presence of exogenous nutrients. The alphaproteobacterium Rhodospirillum centenum is a photosynthetic member of the *Azospirillum* clade, members of which have a life cycle that includes a variety of morphotypes (Fig. 1) (1–3). Replicative vegetative cells of this species are either swim cells with a single sheathed polar flagellum or swarm cells that are peritrichously flagellated, based on environmental viscosity (4, 5). When vegetative cells face carbon starvation or high levels of nitrogen, they differentiate into thick-walled dormant cysts that are desiccation and UV resistant. Cysts can remain dormant for long periods until there is a return of favorable growth conditions (1–3).

For several decades, our laboratory has analyzed cyst formation by R. centenum under aerobic heterotrophic conditions (2, 6–11). During these studies, we observed that wild-type (WT) cells form smooth colonies when grown on nutrient-rich medium that does not induce cyst development and rugose (wrinkled) colonies when grown on cyst-inducing medium containing elevated nitrogen content (7, 8). A linkage of rugose colony formation with cyst development is based on observations that mutants defective in cyst development have never been observed to produce rugose colonies even when grown on cyst-inducing medium (7, 8). Conversely, mutants that are locked in the cyst developmental cycle constantly form rugose colonies replete with cysts even when grown in medium that supports vegetative growth (7, 8).

In general, rugose colonies are thought to be characteristic of biofilm formation, indicating that the formation of a biofilm has a role in the cyst developmental cycle (12, 13). Biofilm formation as a component of cyst development is also consistent with observations of biofilms formed by cells grown in the dark aerobically in cyst-inducing liquid minimal medium. Under this condition, encystation is preceded by pellicle formation, another characteristic feature of biofilms (14). Additionally, transcriptomic analysis of expression changes during cyst development showed a 3- to 8-fold increase in the expression of numerous genes involved in exopolysaccharide (EPS) biosynthesis (11). EPS matrices have been characterized as the “house of biofilm cells” (15) and are a key element in the establishment and maintenance of biofilms (16).

Recently, we have also initiated analysis of the germination phase of the *R. centenum* cyst developmental life cycle (17). This study defined different stages of germination based on microscopic and genome-wide transcription changes. Early notable changes occurred in the expression of genes involved in protein synthesis and the generation of energy through the formation of membrane potential and the synthesis of ATPase. These early expression changes are followed by changes in membrane/cell wall biosynthesis and then by large-scale changes in central metabolism.

Still to be defined are triggering events leading to the initiation of *R. centenum* cyst germination. Many species of *Bacillus* and *Clostridium* utilize highly niche-specific germinants, along with cognate germinant receptors, to initiate spore germination. Germinants are generally defined as small molecules that cue an organism that it is in the right environment for propagation. For example, uric acid present in soil and poultry litter triggers the germination of Bacillus fastidiosus spores, a species that commonly inhabits poultry litter (18). Clostridium difficile spores sense bile salts as strong germinants as their presence informs these cells of an intestinal environment (19, 20). Thus, an organism typically utilizes specific germinants that inform cells that a specific environmental niche is conducive for growth and survival.

In this study, we have assessed what environmental cues trigger the germination of *R. centenum* cysts. During this analysis, we noticed that cyst germination is accompanied by concurrent biofilm/pellicle disintegration. We further observed that biofilm/pellicle disintegration occurs whenever these photosynthetic cells are exposed to light (21). Analyses of different spectra of light, and various deletion mutants, demonstrate that excitation of the photosystem with far-red light is an absolute requirement for the germination of *R. centenum* cysts irrespective of nutrient conditions. Additionally, we show that carbon and nitrogen fixation, which utilize ATP and reducing energy from photosynthesis, are not needed to initiate and complete germination.

## RESULTS

### Biofilm disintegration is a robust visual assay for cyst germination.

**FIG 2:**
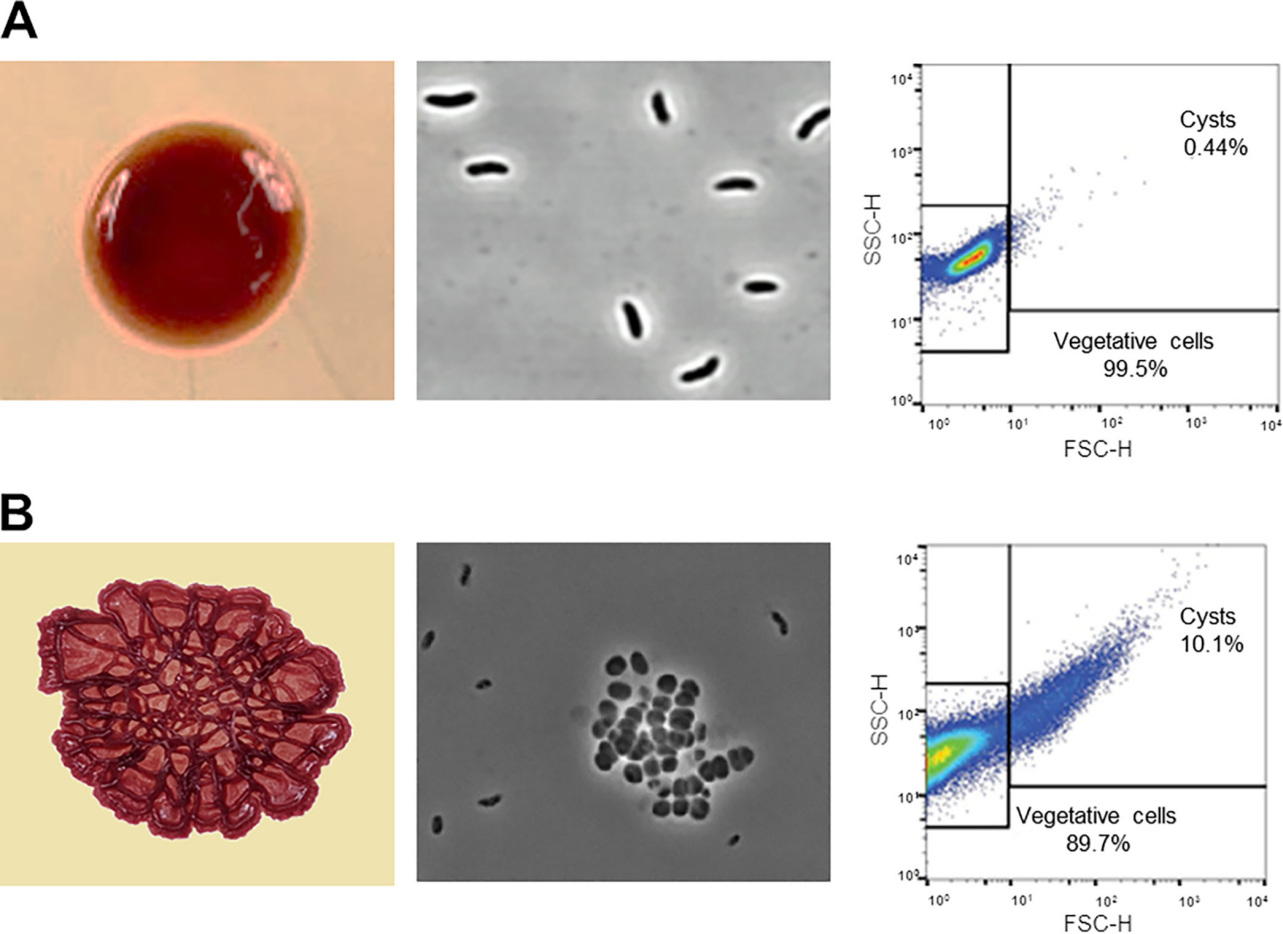
Representative images of WT *R. centenum* cells grown on vegetative CENS and cyst-inducing CENS 8×N media. (A) Smooth colony morphology (left), microscopic analysis of cells in the smooth colony (middle), and flow cytometry quantification of the number of cysts in colonies grown in CENS medium (right). (B) Rugose/wrinkled colony morphology characteristic of biofilms with the corresponding microscopic and flow cytometry analyses of cysts when grown on CENS 8×N solid medium. SSC, side scatter; FSC, forward scatter.

Figure 2A shows typical smooth colony morphology that forms when *R. centenum* cells are grown on agar-solidified CENS medium, which supports vegetative growth. Microscopic and flow cytometry analyses confirm that the vast majority of cells in the colony are in a vegetative state. This is contrasted by the formation of rugose colonies with well-defined ridges and valleys that develop from cells grown in cyst-inducing CENS 8×N medium that contains 8-fold elevated nitrogen content (Fig. 2B) (2, 8). Quantitative flow cytometry analyses show that rugose colonies typically have ∼10% of their cells in a cyst state (Fig. 2B). Previous studies with *R. centenum* cyst developmental mutants have also indicated that rugose colonies are a defining feature of cyst formation. For example, mutants that are defective in cyst formation produce smooth colonies on both vegetative CENS and cyst-inducing CENS 8×N media (2, 8). In contrast, mutants locked in the cyst developmental pathway form rugose colonies on both vegetative CENS and cyst-inducing CENS 8×N media (2, 8). This indicates that the observed rugose colony phenotype is, at least in part, a product of the cyst developmental cycle.

### Light is necessary and sufficient for germination of *R. centenum* cysts.

During our germination analyses, we noticed that the disintegration of cyst-containing biofilms was concurrent with cyst germination as assayed by microscopy and flow cytometry. This allowed the use of biofilm disintegration as a facile visual indicator of cyst germination. We also observed that germination reproducibly occurred in nutrient-rich CENS and CENMED media only when biofilms were exposed to overhead lights or when isolated cysts were exposed to microscopic lights for extended periods during the filming of cyst germination.

**FIG 3:**
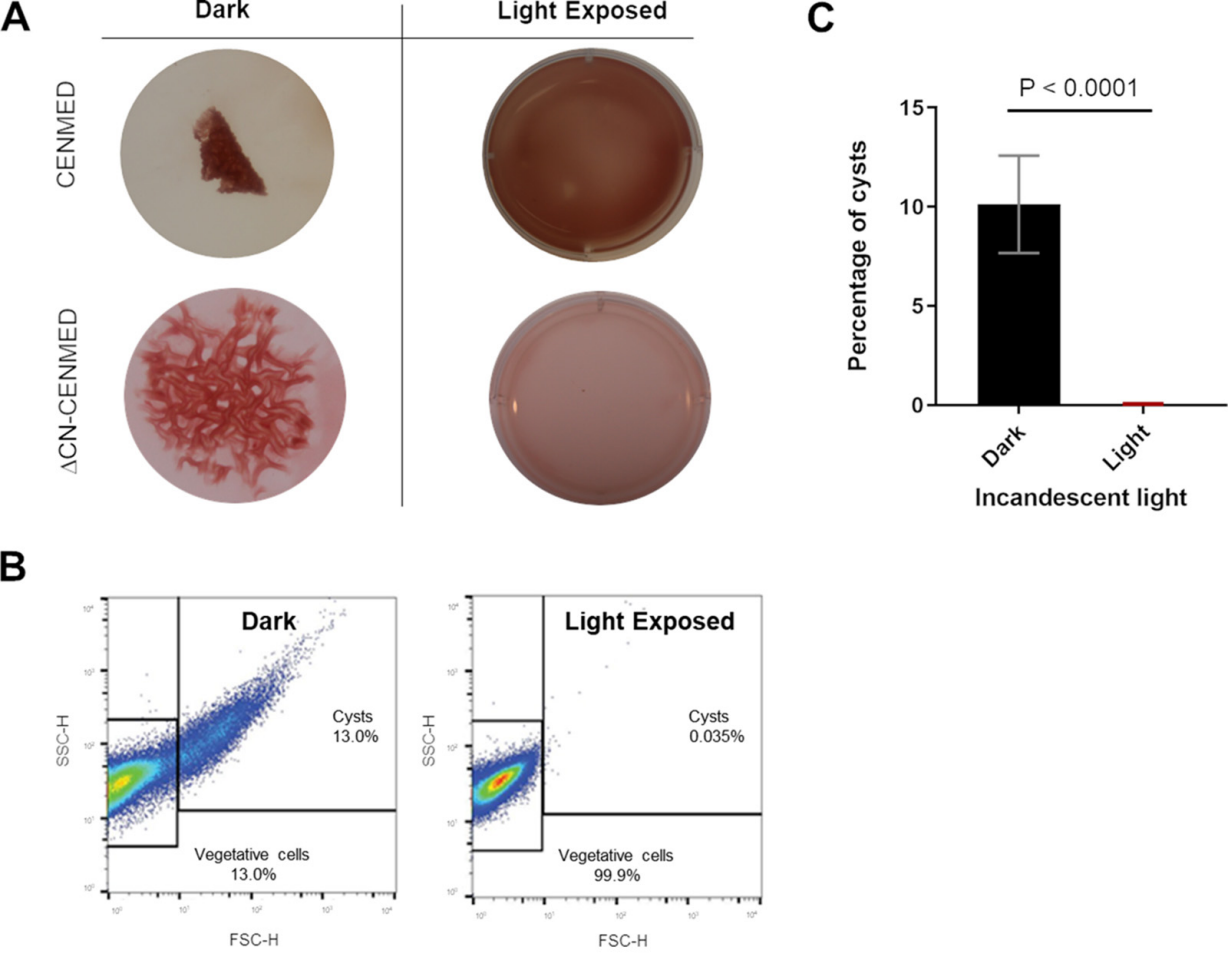
Requirement and sufficiency of light for cyst germination. (A) Biofilm architecture of wild-type *R. centenum* rugose colonies that were suspended and incubated for 24 h in nutrient-rich CENMED or carbon- and nitrogen-free ΔCN-CENMED medium either in the dark or exposed to ∼1,000 mol · m^−2^ · s^−1^ white light. (B) Representative flow cytometry plots from cells collected from rugose colony biofilms suspended in ΔCN-CENMED medium after 24 h of incubation in the dark or with exposure to light. Note the presence of cysts in the cells from biofilms incubated in the dark form a conspicuous comet tail on the data plot that is absent after exposure to light. (C) Percentage of cysts in biofilms incubated in the dark relative to those exposed to incandescent light as quantitated from four biological replicates using flow cytometric analysis. Error bars indicate standard deviations. The bar above the samples indicates the *P* value using an unpaired *t* test. The calculated *P* value compares the mean percentages of cysts between the sample that was light exposed (5 replicates) and the sample that was incubated in the dark (5 replicates).

We subsequently tested whether light was either a germinant or a cogerminant by suspending rugose colony biofilms of wild-type *R. centenum* in either nutrient-complete CENMED medium or ΔCN-CENMED medium, which is CENMED medium devoid of all carbon and nitrogen components. These biofilm suspensions were then incubated in the dark or illuminated with white light (∼1,000 mol · m^−2^ · s^−1^) and visually assessed for biofilm degradation as well as cyst germination via microscopy and flow cytometry. As shown in Fig. 3, biofilm degradation/cyst germination occurred when suspended biofilms were incubated in the light in either nutrient-complete CENMED medium or carbon- and nitrogen-depleted ΔCN-CENMED medium. In contrast, no germination/biofilm degradation occurred when these biofilms were incubated in the dark with or without nutrients. The failure of cysts to undergo germination in the dark even when suspended in nutrient-complete CENMED medium indicates that nutrients alone are not a trigger for germination. Indeed, light exposure was observed to be necessary for germination even when cysts were exposed to nutrients. Furthermore, the observation that cysts effectively germinated in the light even in carbon- and nitrogen-free ΔCN-CENMED medium also demonstrates that light is by itself sufficient for germination.

### Photoreceptors of *R. centenum* are not responsible for light-induced cyst germination.

**FIG 4:**
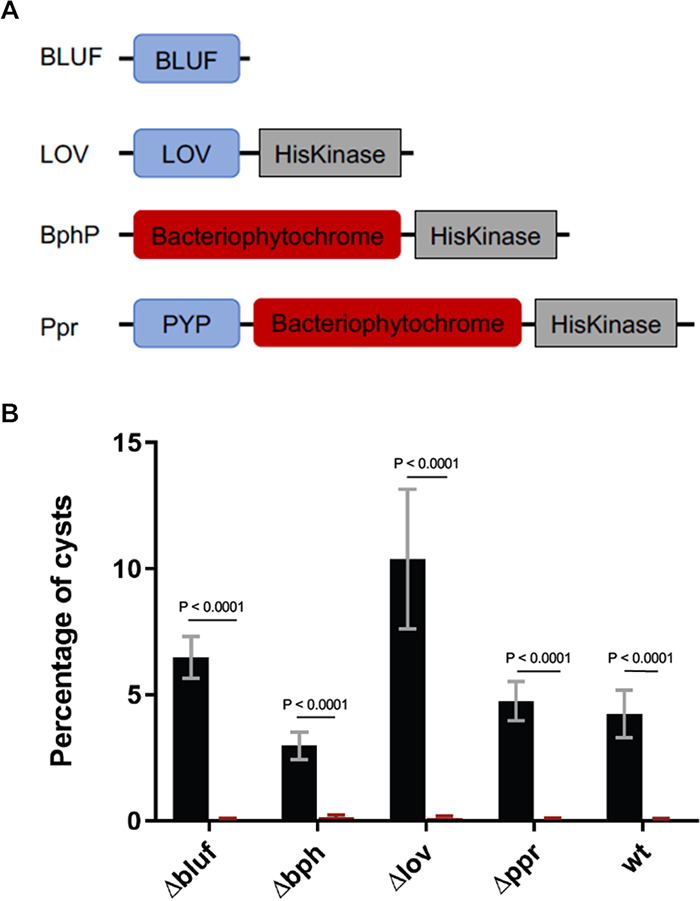
*R. centenum* photoreceptors and their lack of involvement in cyst germination. (A) Domain architecture of all annotated photoreceptors in the *R. centenum* genome. (B) Percentage of cysts in biofilms floating in ΔCN-CENMED medium after incubation in the dark (black bars) versus those exposed to incandescent light (red bars) from wild-type cells and 4 clean deletion mutants of *R. centenum* as measured using flow cytometric analysis. Error bars represent standard deviations obtained from 5 biological replicates. The bars above the samples compared indicate the *P* values determined using an unpaired *t* test. The calculated *P* value compares the mean percentages of cysts between the sample that was light exposed (5 replicates) and the sample that was incubated in the dark (5 replicates) for each strain. In all the mutants, as in the case of the WT, light exposure promoted cyst germination and subsequent biofilm disintegration.

The sequenced and annotated *R. centenum* genome shows the presence of four genes that code for light-sensing photoreceptors (Fig. 4A) (22). RC1_2193 codes for a protein with a putative BLUF (single blue-light-utilizing flavin adenine dinucleotide) domain, while RC1_0351 codes for a histidine kinase that contains a LOV (light-oxygen-voltage) sensory domain that also utilizes flavin for blue light sensing. Additionally, RC1_3803 codes for a bacteriophytochrome (BPh) kinase that likely uses phytochromobilin for red light sensing. Finally, RC1_3224 codes for a hybrid photoreceptor called Ppr, the first bacteriophytochrome identified in proteobacteria (23), which has both a PYP domain that uses *p*-coumaric acid for blue light sensing and a bacteriophytochrome kinase domain that uses phytochromobilin for red light sensing (Fig. 4A). The flavin binding LOV and *p*-coumaric acid binding domains are also members of the Per-Arnt-Sim (PAS) domain family, members of which are also found in several germination receptors of *Bacillales* (24).

We subsequently tested whether any of the annotated photoreceptors are involved in light-mediated rugose biofilm dispersal/cyst germination by constructing individual clean deletions of each of these photoreceptors (Δ*bluf*, Δ*bph*, Δ*lov*, and Δ*ppr*). Cyst-containing rugose colonies formed by these deletion strains were lifted off plates and incubated in liquid ΔCN-CENMED medium in the dark or illuminated with incandescent white light. All the resuspended photoreceptor mutant biofilms underwent dispersal similar to that of wild-type biofilms upon illumination (Fig. 4B). This indicates that the tested photoreceptors are not responsible for light-induced germination of *R. centenum* cysts. This is unlike the nonphotosynthetic bacterium Ramlibacter tataouinensis, where the switch between the cyst and vegetative states is controlled by photoreceptors (25).

### Far-red light that drives photosynthesis also induces cyst germination and biofilm disintegration.

**FIG 5:**
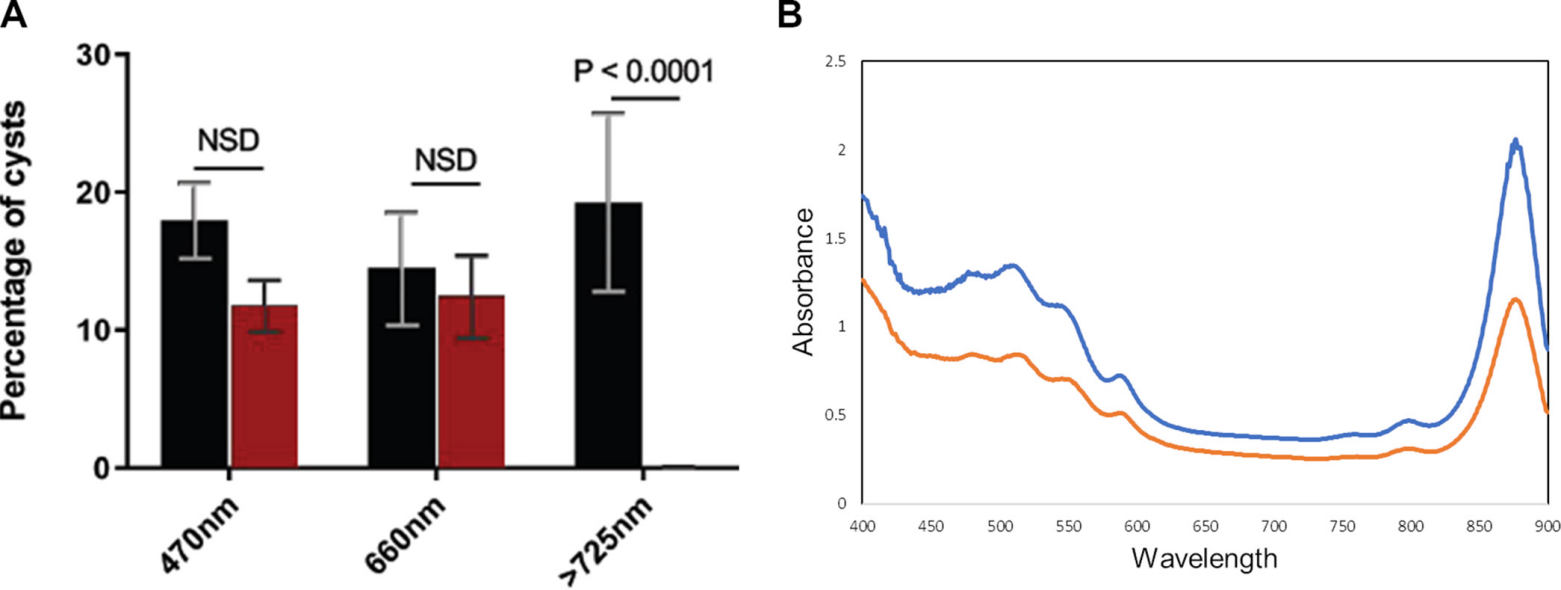
Effect of light wavelength on cyst germination. (A) Percentages of cysts in WT biofilms kept in the dark (black bars) and those exposed to monochromatic blue light (470 nm), monochromatic red light (660 nm), and far-red light (wavelengths beyond 725 nm) as assayed by flow cytometric quantification. Error bars represent standard deviations obtained from 4 biological replicates. The bars above the samples compared indicate the *P* values determined using an unpaired *t* test. The calculated *P* value compares the mean percentages of cysts between the WT sample that was exposed (4 replicates) to light greater than 725 nm and the sample that was incubated in the dark (4 replicates). NSD indicates no significant statistical difference between the samples when analyzed by an unpaired *t* test. (B) Spectrophotometric scan of purified cysts (blue) and vegetative cells (orange) of *R. centenum*. The peaks marked show the absorbance wavelengths of all the light receptors. Beyond 730 nm, the peaks show the absorbances of bacteriochlorophyll and the photosynthetic reaction center.

We next addressed which wavelengths of light promote cyst germination by exposing cysts to different wavelengths of light. For this analysis, we resuspended wild-type rugose colonies in carbon- and nitrogen-free ΔCN-CENMED medium and illuminated aliquots with monochromatic blue light (470 nm), monochromatic red light (660 nm), or white light filtered through a long-band-pass filter that allows only far-red light beyond 725 nm to pass through. Wild-type rugose biofilms illuminated with monochromatic blue light or monochromatic red light exhibit neither biofilm disintegration nor cyst germination (Fig. 5A). In contrast, exposure of rugose biofilms to far-red light beyond 725 nm led to visual biofilm disintegration and cyst germination as assayed by flow cytometry (Fig. 5A). This result also supports our conclusion that photoreceptors are not involved in cyst germination as they typically absorb light in the visible spectrum below 700 nm (Fig. 5B).

**FIG 6:**
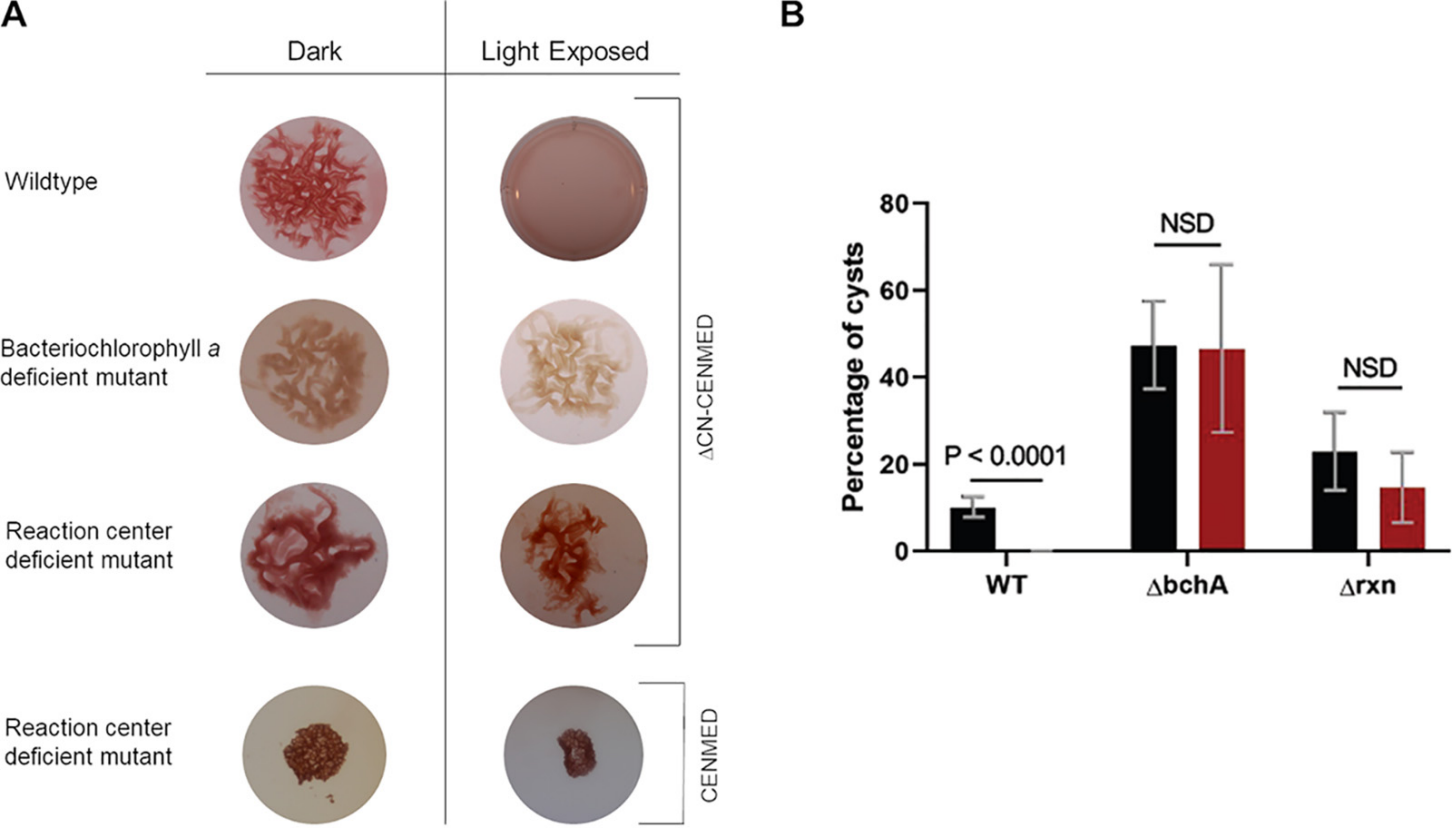
A functional photosystem is required for cyst germination. (A) Rugose colony biofilm architecture of the wild type, a bacteriochlorophyll *a*-deficient mutant, and a reaction center-deficient mutant that were incubated in the dark or exposed to incandescent white light. The top three rugose colony biofilms were suspended in carbon- and nitrogen-free ΔCN-CENMED medium, while the bottom reaction center rugose colony biofilm was suspended in nutrient-rich CENMED medium. (B) Percentages of cysts from cells collected from the wild-type, bacteriochlorophyll *a*, and reaction center biofilms in carbon- and nitrogen-free ΔCN-CENMED medium under dark and illuminated conditions as determined by flow cytometry. Black bars show samples incubated in the dark, and red bars show samples exposed to incandescent light. Error bars represent standard deviations obtained from 3 biological replicates. The bars above the samples compared indicate the *P* values determined using an unpaired *t* test. The calculated *P* value compares the mean percentages of cysts between the wild-type sample that was exposed (4 replicates) to light and the sample that was incubated in the dark (4 replicates). NSD indicates no significant statistical difference between the samples when analyzed by an unpaired *t* test.

Spectral analyses of vegetative and cyst cells (Fig. 5B) show that both of these cell types harbor photopigment peaks at 800 and 875 nm that are characteristic of the previously characterized *R. centenum* photosystem (3, 21, 26). This confirms the results of a transcriptomic analysis that indicates that photosystem genes do not undergo significant changes in expression as cells transition from vegetative to cyst forms (11). To test whether light excitation of the photosystem induces cyst germination, we grew cyst-containing rugose colonies on 8×N CENS plates from a mutant strain that could not make bacteriochlorophyll *a* (Δ*bchA*) as well as from a mutant that could make photopigments but did not produce a functional reaction center (Δ*rxn*) as needed for photosynthesis (27). As shown in Fig. 6, rugose colonies from the photosynthesis-defective mutants were not capable of disintegration or cyst germination when illuminated after resuspension in carbon- and nitrogen-free ΔCN-CENMED medium. The same photosynthesis-deficient strains were also defective in biofilm disintegration/cyst germination when suspended in nutrient-rich CENMED medium both in the dark as well as under illuminated conditions.

Finally, the failure of these photosynthesis-defective mutants to germinate was also directly visualized by placing cysts in agarose-solidified CENS medium on an illuminated microscopic slide. In this setup, the microscope’s light was always on, with images recorded every 5 to 10 min. As observed in Movie S1A and Movie S1B in the supplemental material, wild-type cysts germinated over a 6-h period, while cysts derived from the photosynthesis-deficient reaction center mutant failed to exhibit any evidence of germination. Collectively, these results establish that light excitation of the photosystem is both necessary and sufficient for cyst germination.

10.1128/mBio.03619-20.3 MOVIE S1A Time-lapse video of wild-type cysts germinating in CENS medium. Both cell types were incubated under a microscope under similar conditions with the microscope’s light left on during the 12-h imaging period. Download Movie S1A, AVI file, 7.3 MB. Copyright © 2021 Ashok et al. 2021 Ashok et al. https://creativecommons.org/licenses/by/4.0/ This content is distributed under the terms of the Creative Commons Attribution 4.0 International license.

10.1128/mBio.03619-20.4 MOVIE S1B The Δ*rxn* reaction center mutant, which is incapable of photosynthesis, is defective in germination in CENS medium. Both cell types were incubated under a microscope under similar conditions with the microscope’s light left on during the 12-h imaging period. Download Movie S1B, AVI file, 4.6 MB. Copyright © 2021 Ashok et al. 2021 Ashok et al. https://creativecommons.org/licenses/by/4.0/ This content is distributed under the terms of the Creative Commons Attribution 4.0 International license.

### Carbon and nitrogen fixation are not needed for cyst germination.

**FIG 7:**
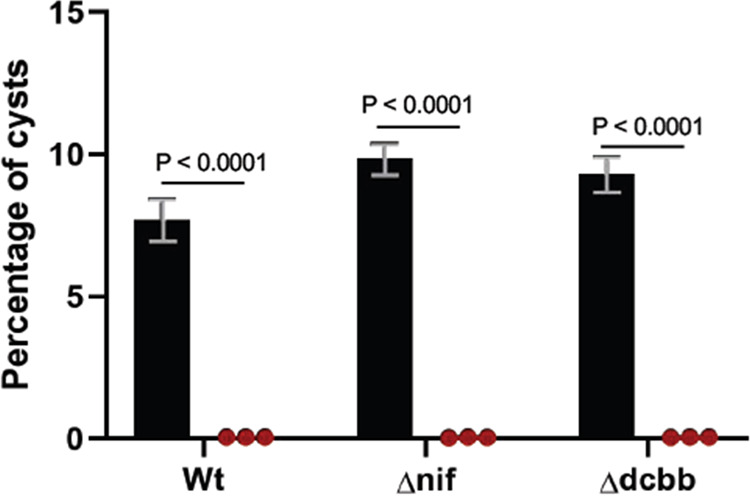
Light-driven germination does not require carbon or nitrogen fixation. (A) Percentages of cysts in rugose colony biofilms suspended in carbon- and nitrogen-free ΔCN-CENMED medium from wild-type cells, a strain that has both *cbb* gene clusters deleted (Δdcbb), and a strain that has the *nifHDK* operon deleted (Δnif), incubated in dark (black bars) and exposed to ∼1,000 mol · m^−2^ · s^−1^ white light (red bars). The percentages of cysts were calculated using flow cytometry. Error bars represent standard deviations obtained from 3 biological replicates. The bars above the samples compared indicate the *P* values determined using an unpaired *t* test. The calculated *P* value compares the mean percentages of cysts between the sample that was light exposed (3 replicates) and the sample that was incubated in the dark (3 replicates) for each strain.

We also tested if photosynthesis may be providing energy for downstream carbon and/or nitrogen fixation pathways that may form a product that subsequently triggers germination. For this analysis, we constructed strains that were defective in carbon or nitrogen fixation and tested whether they are capable of undergoing cyst germination. *R. centenum* has two sets of RUBISCO genes, the form IC *cbb* operon and the form IAq *cbb* operon, both of which can drive CO_2_ fixation (22). A double-deletion strain (Δd*cbb*) that lacked both these *cbb* operons was therefore constructed. As shown in Fig. 7, cysts from the Δd*cbb* strain germinate at levels equivalent to those of WT cysts in carbon- and nitrogen-free ΔCN-CENMED medium, thereby indicating that carbon fixation is not needed for light-driven germination.

Photosynthetic organisms also use energy from photosynthesis for driving nitrogen fixation in the absence of inorganic nitrogen sources. Because several amino acids have been identified as triggers of spore germination, we also tested if nitrogen fixation is needed for cyst germination. For this analysis, we constructed a strain that lacked the nitrogenase operon of *R. centenum* (Δ*nif*) and likewise observed that cysts derived from this strain also germinated on par with WT cysts when exposed to light in carbon- and nitrogen-free ΔCN-CENMED medium (Fig. 7). This indicates that fixed nitrogen is also not a requirement for germination in response to far-red light excitation of the photosystem.

## DISCUSSION

Germinants are typically small extracellular molecules that signal dormant cells to initiate the germination process when conditions are favorable for growth and propagation. The chemical nature of germinants varies among species based on the niche that a specific organism occupies. For example, Bacillus fastidiosus, which thrives in poultry litter, uses uric acid found in the excretory product of birds as its only spore germinant (18). Bacillus anthracis germinates only when exposed to nutrients associated with phagocytes (28) and requires a nucleoside-amino acid combination or two different amino acids to germinate (29–32). Clostridium difficile spores germinate when their environment resembles that of a mammalian gut; i.e., this species needs bile salts to trigger germination as well as amino acids such as glycine as a cogerminant (20, 28, 33, 34). Thus, the nature of a microorganism’s germinant provides information regarding what the microbe considers an environment favorable for growth.

*R. centenum* is a metabolically flexible photosynthetic organism that can use a variety of energy sources for growth (22). Under light conditions, these cells can grow photoheterotrophically, where they derive energy from both photosynthesis and a carbon source. They can also grow photoautotrophically, where they obtain all of their energy from photosynthesis and use this energy to drive both carbon (CO_2_) and nitrogen (N_2_) fixation to feed cellular metabolism. Under dark growth conditions, these cells can also grow heterotrophically using an exogenous carbon source for metabolism and energy production. The metabolic flexibility of these cells, coupled with their ability to grow under dark conditions, would suggest that cysts should be capable of cuing in to a wide variety of signals to induce germination. However, this does not appear to be the case as *R. centenum* cysts did not germinate when incubated in a nutrient-complete medium under dark conditions. This indicates that *R. centenum* cysts are not assessing environmental nutrient availability as a cue to initiate germination. The fact that cysts have an absolute requirement for excitation of the photosystem in order to germinate indicates that photosynthetic growth capability is the environmental condition that these cells deem essential for emergence from dormancy. Finally, the observation that cysts from Δd*cbb* and Δ*nif* deletion constructs undergo normal light-dependent germination also suggests that these cells are not using photosynthesis as an energy source to fix carbon and nitrogen into an organic compound that is subsequently functioning as a germinant. While we cannot rule out that cysts may be using energy from photosynthesis to drive metabolic remodeling of internal storage compounds such as polyhydroxybutyrate (PHB) or amino acid pools into an organic germinant cue, it is intriguing to consider that the germinant signal that drives cyst germination in this species may be the photosynthesis production of energy in the form of reduced ubiquinone or the downstream generation of NADH or ATP. If this is the case, then there may be an internal germinant receptor that senses the input of energy by photosynthesis into one or more of these pools, which then cues the germination pathway. Indeed, there are examples of histidine sensor kinases that monitor the redox state of the ubiquinone pool (35–39) as well as sensor kinases that regulate kinase and/or phosphatase activity in response to a cell’s energy charge, as denoted by ATP/ATP plus ADP (6, 40–42). A reduction in ATP production has been shown to affect the activity of the sensor kinase CheS, resulting in the induction of cyst formation (6). It would be notable if the photosynthesis-driven increase in ATP production were to lead to cyst germination. However, the mechanism by which photosynthesis induces cyst germination will have to wait for additional studies.

Finally, the nonphotosynthetic betaproteobacterium Ramlibacter tataouinensis has two red-light-sensitive bacteriophytochromes that regulate the switch between rod-shaped vegetative cells and desiccation-resistant cyst cells. In this species, darkness favors cyst formation, while far-red light favors vegetative cell formation (25). Therefore, we addressed whether light excitation of photoreceptors could also function as a germination signal for *R. centenum*. However, none of the tested photoreceptors, nor light in the visible spectrum that these photoreceptors likely sense, affected *R. centenum*’s cyst germination.

Taking these results into consideration, we now know that this organism’s life cycle (Fig. 1) (i) involves the induction of cyst formation under high-nitrogen/poor-carbon conditions, (ii) couples cyst formation with the formation of a biofilm, (iii) couples cyst germination with biofilm disintegration, and (iv) exits dormancy only when generating energy from photosynthesis. To our knowledge, this is the first example where germination is dependent on a nonchemical environmental energy signal.

## MATERIALS AND METHODS

### Bacterial strains, growth conditions, and biofilm/cyst induction.

Table S1 in the supplemental material shows a complete list of strains used in this study. Wild-type (ATCC 51521) and mutant strains of *R. centenum* were routinely grown and maintained on CENS solid medium with an incubation temperature of 37°C. CENS medium is a modification of the previously described CENMED medium (3) by the addition of 4 g/liter of Bacto Soytone. ΔCN-CENMED medium is a carbon- and nitrogen-free derivative of CENMED medium from which sodium pyruvate, ammonium chloride, disodium EDTA, vitamin B_12_, and biotin were omitted.

10.1128/mBio.03619-20.1 TABLE S1 Strains used in this study. Download Table S1, PDF file, 0.04 MB. Copyright © 2021 Ashok et al. 2021 Ashok et al. https://creativecommons.org/licenses/by/4.0/ This content is distributed under the terms of the Creative Commons Attribution 4.0 International license.

For the induction of cysts, single colonies from plates were grown aerobically in liquid CENS medium overnight at 37°C. Fifty microliters of the culture grown overnight was subcultured into 5 ml of fresh liquid CENS medium and grown aerobically at 37°C for 15 to 16 h. The culture was then adjusted to an optical density at 660 nm (OD_660_) of 2.0 either by centrifugation and resuspension into smaller volumes of CENS medium or by dilution with fresh medium. One milliliter of the culture at an OD_660_ of 2.0 was then washed three times in 40 mM potassium phosphate buffer (pH 7.5) and resuspended in 50 μl of the same buffer. Five-microliter inoculation spots were then placed on sterile 0.45-μm nylon membranes that were then placed onto air-dried CENS 8×N plates (CENS 8×N medium is the same as CENS medium with 8 times the ammonium chloride concentration [7]). The plates were then incubated in the dark at 37°C for 48 to 60 h to allow the development of cyst-containing rugose colonies. For spectral analysis of cysts, the cysts were separated from vegetative cells (purified) using lysozyme disruption as described previously (17).

### Generation of mutant strains.

The construction of photoreceptor gene deletions (Δ*ppr*, Δ*lov*, Δ*bluf*, and Δ*bph*) was previously described (43). Clean deletions were performed for the remaining genes, the *nifHDK* gene cluster, and construction of a double-RUBISCO mutant (Δd*cbb*). The latter was performed by first deleting the form IC *cbb* operon and the form IAq *cbb* operon using a previously reported markerless double-recombination technique (44, 45). In short, gene fragments of approximately 1 kb in length containing ∼500 bp of DNA flanking the targeted gene were amplified by crossover PCR with primers listed in Table S2. The PCR products were sequenced and subcloned into the gentamicin (Gm)-resistant suicide vector pZJD29a that also harbors a *sacB* gene providing sucrose sensitivity for plasmid counterselection. The resulting constructs have flanking regions and a short 6-amino-acid reading frame that replaces the original gene of interest. Suicide plasmids containing the correct sequenced insertions were transformed into Escherichia coli S-17(λpir) for conjugal mating with *R. centenum*. For mating, the E. coli strain was grown in LB medium with 10 μg/ml gentamicin to exponential phase, at which point 750 μl was harvested and washed 2 times with fresh CENS medium. The resulting pellet was resuspended in 750 μl of an *R. centenum* culture grown overnight (CENS medium) and pelleted again. The supernatant was removed, and cells were then resuspended in 50 μl CENS medium and spotted onto CENS plates without antibiotics. The plates were incubated for 6 h at 37°C, after which the cells were streaked onto selective medium (CENS medium with 25 μg/ml kanamycin and 10 μg/ml gentamicin). Colonies were visible on the selective plates after incubation for 2 days at 42°C. Several colonies were then restreaked onto selective medium and subsequently onto CENS medium with 5% sucrose. Mutants were screened for double-crossover recombination events by screening for gentamicin-sensitive colonies by replicative patching on both CENS medium and CENS medium containing 10 μg/ml gentamicin. The genotypes of the mutants were subsequently confirmed by colony PCR amplification and sequencing.

10.1128/mBio.03619-20.2 TABLE S2 Primers used to construct suicide plasmids. Download Table S2, PDF file, 0.07 MB. Copyright © 2021 Ashok et al. 2021 Ashok et al. https://creativecommons.org/licenses/by/4.0/ This content is distributed under the terms of the Creative Commons Attribution 4.0 International license.

### Light-induced cyst germination/biofilm disintegration assays.

Wrinkled rugose biofilm-containing colonies were grown on 0.45-μm nylon membranes placed over agar-solidified CENS 8×N plates. For resuspension of rugose colonies in buffer, the membrane filters containing a rugose colony were lifted from the agar plate and placed onto the surface of a petri dish containing liquid ΔCN-CENMED, CENMED (3), or CENS medium. Within 2 to 5 min, the rugose colonies cleanly floated away from the membrane without losing structural integrity. The floating rugose colony biofilms were then exposed to either 1,000 μmol · m^−2^ · s^−1^ of white light emitted from a 60-W incandescent light source, monochromatic blue light-emitting diode (LED) light (470 ± 24 nm), or red LED light (660 ± 19 nm) for 24 h at room temperature. A far-red light filter that filters wavelengths beyond 725 nm was used for far-red light illumination by placement of the filter in front of a 60-W incandescent light. Wrinkled rugose colonies in carbon- and nitrogen-free buffer, CENS medium, or CENMED medium were also incubated in the dark as controls.

### Microscopic germination assay.

Rugose colonies from CENS 8×N plates were resuspended in 40 mM phosphate buffer (pH 7) and briefly (1 to 3 s) disrupted by sonication. One microliter of the sonicated biofilms was placed on top of a CENS pad (1% agarose) on a concavity slide with a coverslip placed over the concave region and sealed using Vaseline-lanolin-paraffin (46). The slide was incubated on a 60× oil immersion objective of a Nikon TiE inverted microscope. The slide was enclosed within a stage-top incubator, which maintained the slide at 37°C. Cysts were located, the *x* and *y* coordinates were recorded, and the microscope was set to image every 10 min for ∼24 h. The microscope light was left on during the entire time to ensure that cysts were exposed to light. ImageJ (Fiji) was used to add timestamps and for stitching the images into a movie.

### Quantification of cyst germination/biofilm disintegration.

The amounts of vegetative and cyst cells in a biofilm or growth culture were quantitatively measured by flow cytometry analysis. Briefly, after periods of dark or light incubation, 1-ml samples were prepared for flow cytometry analysis by disruption of residual biofilm via brief (3 to 4 s) sonication using a Branson 450 sonifier with the duty cycle set to constant and the output control set to 1 for three to four 1-s bursts. Each 1-ml sample was then stained with 0.1 μl of 5 mM Syto 9 and incubated in the dark for 30 min before running samples on a FACSCalibur or a MACSQuant VYB instrument with 100,000 cells/events run for each sample. Cysts, due to their larger size and higher internal complexity, have higher forward and side scattering of light than vegetative cells (10). Due to this difference from vegetative cells, cysts form a comet-tail-like structure on a flow cytometry plot, while vegetative cells form a compact cluster near the axis (10). The percentages of vegetative and cyst cells were determined by gating using FlowJo software. A sample comprised of vegetative cells was run on a FACSCalibur/MACSQuant VYB instrument and used as a control for gating. FlowJo software automatically calculates the percentages of cysts and vegetative cells in each sample after gating.
