# Supplementary material for: No Light, No Germination: Excitation of the Rhodospirillum centenum Photosynthetic Apparatus Is Necessary and Sufficient for Cyst Germination
Source: mBio. 2021 Mar 16;12(2):e03619-20. doi: 10.1128/mBio.03619-20 (PMC8092318; doi:10.1128/mBio.03619-20)
Supplement: TABLE S1 [file mBio.03619-20-st001.pdf]

Supplemental Table S1: Strains used in this study.

Species	*Strain*	*Reference*
Rhodospirillum centenum	*Wild type*	*ATCC51521*
Rhodospirillum centenum	*Δbluf*	*This study*
Rhodospirillum centenum	*Δluv*	*This study*
Rhodospirillum centenum	*Δbph*	*This study*
Rhodospirillum centenum	*Δppr*	*This study*
Rhodospirillum centenum	*Δrxn*	*(27)*
Rhodospirillum centenum	*ΔbchA*	*(27)*
Rhodospirillum centenum	*Δdcbb*	*This study*
Rhodospirillum centenum	*Δnif*	*This study*
