# Supplementary material for: No Light, No Germination: Excitation of the Rhodospirillum centenum Photosynthetic Apparatus Is Necessary and Sufficient for Cyst Germination
Source: mBio. 2021 Mar 16;12(2):e03619-20. doi: 10.1128/mBio.03619-20 (PMC8092318; doi:10.1128/mBio.03619-20)
Supplement: TABLE S2 [file mBio.03619-20-st002.pdf]

Supplemental Table S2: Primers used to construct suicide plasmids.

**Target Gene**	**Primer Name**	**Primer Sequence (5'-3')**
lov	1lov_sacIf	GGCGCTGAGCTCCGACGACATCGATGTCGAGGG
lov	2lov_crossr	TCAACGACGAGAATCGTGGCCCAGCATCTCCCGCTCCCGCAT
lov	3lov_crossf	ATGCGGGAGCGGGAGATGCTGGGCCACGATTCTCGTCGTTGA
lov	4lov_xbaIr	AATTCCCGCAGCAGGCGCGCCTCTAGACCGTGT
bluf	1bluf_sacIf	CTGGGCGAGCTCGACGCCCAACCCTGGAGGA
bluf	2bluf_crossr	CTACACCTCCACGAGGTTGTCGAGCAGCGCGTCGTCGGTCAT
bluf	3bluf_crossf	ATGACCGACGACGCGCTGCTCGACAACCTCGTGGAGGTGTAG
bluf	4bluf_xbaIr	GGGCGGTCTAGAGCTCGACCTGTCCGCGATC
bphP	1bphp_sacIf	CTATGTGAGCTCTCTCGTCCGCGGGATGGTC
bphP	2bphp_crossr	TCAGCCGTGCCATTGTAGCCGGACCTGGTCAATCACGTCCAC
bphP	3bphp_crossf	GTGGACGTGATTGACCAGGTCCGGCTACAATGGCACGGCTGA
bphP	4bphp_xbaIr	GCGGCCTCTAGAAAGGCGCGGCGGCATCTCT

IC *cbb*	Δ0239-0240-pcr1-F	AAGCTTGCATGCCTGCAGGTCGACTAGATGCGGCGTGAAATACTTGGCCG
IC *cbb*	Δ0239-0240-pcr1-R	TGCCTGATAGGTCTTGCCCATCCTGTGCTCCTCTTGTCCCGG
IC *cbb*	Δ0239-0240-pcr2-F	CAGGATGGGCAAGACCTATCAGGCACGGGCGCCCGTGGCGCGCTGATCCG
IC *cbb*	Δ0239-0240-pcr2-R	TGTAAAACGACGGCCAGTGAATTCGCGCCCTTCTTGTCCAGGGGCAGCAT
IAq *cbb*	Δ4061-4062-pcr1-F	AAGCTTGCATGCCTGCAGGTCGACTACCCCCGGCCGCTCCGCCTGGAAGC
IAq *cbb*	Δ4061-4062-pcr1-R	ACCGGAAACACGGGTATCCATGGCGACTACCCCTATTGGTTG
IAq *cbb*	Δ4061-4062-pcr2-F	CGCCATGGATACCCGTGTTTCCGGTGGGACGGGAAATGGCCGATAGGCCC
IAq *cbb*	Δ4061-4062-pcr2-F	TGTAAAACGACGGCCAGTGAATTCGCGTCGATGCGGCGGCGGATGGCGGC
nif	Δ3681-3683-pcr1-F	AAGCTTGCATGCCTGCAGGTCGACTCGTGAAGCCGGTGCGGTAGAGCGTC
nif	Δ3681-3683-pcr1-R	GCCTTCGACCTGACGCGATAGGCGTCTTTCCAGCCCGTCTGG

nif	Δ3681-3683-pcr2-F	ACGCCTATCGCGTCAGGTCGAAGGCGCGGAGCTTGTTGCGGGCCATGGGA
nif	Δ3681-3683-pcr2-R	TGTAAAACGACGGCCAGTGAATTCGTGGCCGGGGTGCCCGTCCTGACGGC
